# Covid-19 transmission in fitness centers in Norway - a randomized trial

**DOI:** 10.1186/s12889-021-12073-0

**Published:** 2021-11-16

**Authors:** Lise M. Helsingen, Magnus Løberg, Erle Refsum, Dagrun Kyte Gjøstein, Paulina Wieszczy, Ørjan Olsvik, Frederik E. Juul, Ishita Barua, Henriette C. Jodal, Magnhild Herfindal, Yuichi Mori, Solveig Jore, Fridtjof Lund-Johansen, Atle Fretheim, Michael Bretthauer, Mette Kalager, for the TRAiN study group, Anita Aalby, Madeleine Berli, Siv Furholm, Anne-Lise Horvli, Line Norum, Halvor Lauvstad, Judit Somogyi, Alexander Myers, Tonje Poulsson, Wenche Evertsen, Hilde Sandvoll, Kjersti Oppen

**Affiliations:** 1 Clinical Effectiveness Research Group, University of Oslo and Oslo University Hospital, Oslo, Norway; 2 Institute of Medical Biology, Norwegian Arctic University, Tromsø, Norway; 3 Norwegian Institute of Public Health, Oslo, Norway; 4 Department of Immunology, Oslo University Hospital, Oslo, Norway

## Abstract

**Background:**

Closed fitness centers during the Covid-19 pandemic may negatively impact health and wellbeing. We assessed whether training at fitness centers increases the risk of SARS-CoV-2 virus infection.

**Methods:**

In a two-group parallel randomized controlled trial, fitness center members aged 18 to 64 without Covid-19-relevant comorbidities, were randomized to access to training at a fitness center or no-access. Fitness centers applied physical distancing (1 m for floor exercise, 2 m for high-intensity classes) and enhanced hand and surface hygiene. Primary outcomes were SARS-CoV-2 RNA status by polymerase chain reaction (PCR) after 14 days, hospital admission after 21 days. The secondary endpoint was SARS-CoV-2 antibody status after 1 month.

**Results:**

3764 individuals were randomized; 1896 to the training arm and 1868 to the no-training arm. In the training arm, 81.8% trained at least once, and 38.5% trained ≥six times. Of 3016 individuals who returned the SARS-CoV-2 RNA tests (80.5%), there was one positive test in the training arm, and none in the no-training arm (risk difference 0.053%; 95% CI − 0.050 to 0.156%; *p* = 0.32). Eleven individuals in the training arm (0.8% of tested) and 27 in the no-training arm (2.4% of tested) tested positive for SARS-CoV-2 antibodies (risk difference − 0.87%; 95%CI − 1.52% to − 0.23%; *p* = 0.001). No outpatient visits or hospital admissions due to Covid-19 occurred in either arm.

**Conclusion:**

Provided good hygiene and physical distancing measures and low population prevalence of SARS-CoV-2 infection, there was no increased infection risk of SARS-CoV-2 in fitness centers in Oslo, Norway for individuals without Covid-19-relevant comorbidities.

**Trial registration:**

The trial was prospectively registered in ClinicalTrials.gov on May 13, 2020. Due to administrative issues it was first posted on the register website on May 29, 2020: NCT04406909.

**Supplementary Information:**

The online version contains supplementary material available at 10.1186/s12889-021-12073-0.

## Background

Governments and health policy makers worldwide have been taking preventive measures against Covid-19 (Coronavirus disease of 2019) exceeding previous pandemics [1]. Social distancing such as increased distance between individuals (minimum 1 or 2 m) is of paramount importance to contain spread of Covid-19. Many countries have closed or restricted access to schools, stores, restaurants, and work places to achieve social distancing [2].

Whilst keeping adequate distance between individuals may involve little disturbance for daily life, closures of schools, recreational activities, and work places have potentially large consequences for education, health and wellbeing, and personal and societal economy. Thus, it is important to assess social distancing measures and gain knowledge about their impacts on society as a whole [3]. Due to the uncertainty of contagiousness, immunity, morbidity and mortality of Covid-19, it is unclear how to resume activities without risking increased spread of disease.

Training and exercise is important for health and wellbeing, and fitness centers are important for many individuals, and for population health. In Norway, by governmental emergency law, all fitness centers closed on March 12 to June 15, 2020 [4, 5]. Surveys have indicated that Norwegians have a more sedentary lifestyle and exercise less after the restrictions were implemented [6]. To prevent negative net effects on health, the effectiveness of closing on disease spread must be weighed against impact on society, and unnecessary closings prevented.

We hypothesised that the risk of SARS-CoV-2 (severe acute respiratory syndrome coronavirus 2) transmission in fitness centers would be low, provided strict implementation and enforcement of good hygiene and physical distancing measures. This report describes the incremental risk of SARS-CoV-2 infection through the re-opening of fitness centers with stringent use of mitigation measures during the Covid-19 pandemic in Oslo, Norway, in May and June 2020.

## Methods

This study adheres to the CONSORT (Consolidated Standards of Reporting Trials) guidelines.

### Setting

All fitness centers in Norway closed due to the Covid-19 pandemic on March 12, 2020. For the purpose of the trial, five fitness centers in Oslo opened their premises to participants randomized to training from May 25 to June 15, 2020 (details in Supplementary Appendix). All other fitness centers in the country remained closed, and participants in the no-training control arm did not have access.

### Participants

All members of the participating fitness centers age 18 years or older who were not at increased risk for severe Covid-19 per criteria by the Norwegian Institute of Public Health, were eligible for participation. The criteria for high risk were at least one of the following: age 65 years or older; cardiovascular disease including hypertension; diabetes [7].

Fitness center members were approached by email and those interested signed up for the study through a secure website at the University of Oslo. Co-morbidities were self-assessed. A direct contact telephone line and email address to the study team was established for interested individuals in case of uncertainty of their medical status and other questions. All eligible individuals were informed about the nature of the trial, and provided consent before randomization.

### Randomization and procedures

ML randomized eligible individuals 1:1 stratified by fitness center by a computerized random number generator and assigned participants either to current practice, which was no access (no-training arm) to the fitness center, or to access (training arm) with mitigation measures as described in the “Norwegian guidelines for Hygiene and Social Distancing in Fitness centers during the Covid-19 Pandemic”, available at https://t-i.no/wp-content/uploads/2020/04/Bransjestandard-for-sentre.pdf. ML informed the fitness centers about the participants assigned to the intervention arm. Participants were informed about their assigned study arm by an email from the IT department at the University of Oslo.

The following measures were enforced at all facilities during the trial: Avoidance of body contact; 1 m distance between individuals at all times; 2 m distance for high intensity activities; provision of disinfectants at all work stations; cleaning requirements of all equipment after use by participant; regular cleaning of facilities and access control by facility employees to ensure distance measures and avoid overcrowding. Changing rooms were open, but showers and saunas remained closed. Staff was present during all opening hours. Lids on trash cans were removed. Individuals were instructed to stay home if they had any Covid-19 related symptoms. No masks were required, but members were advised to avoid touching their eyes, nose and mouth.

Activities for the training arm included services the fitness centers provide ordinarily, including fitness floor activities and group classes. The infection preventive measures applied at the participating centers during the study period are detailed in the Supplementary Appendix.

### Outcomes

The primary endpoint was the proportion of SARS-CoV-2 RNA (Ribonucelic acid) positive individuals in the two study arms after 14 to 15 days. Co-primary endpoint was hospital admission in the two arms after 21 days, and secondary endpoint was the proportion of individuals with SARS-CoV-2 antibodies in the two study arms after 30 days.

### SARS-CoV-2 RNA testing

To resemble a situation where fitness centers in general would be allowed to re-open, no participants were tested for Covid-19 before entering the study. All participants were mailed a home-test kit including two swabs and a tube with virus transport medium for SARS-CoV-2 RNA. The tests were analysed with a real-time SARS-CoV-2 RT-PCR (reverse transcription-polymerase chain reaction) test (Cobas®, Roche Diagnostics Inc.) at the Department of Medical Microbiology, Oslo University Hospital. Participants were instructed to sample from the oropharynx, nose and saliva according to national guidelines after median 2 weeks of training access in the training arm (June 8 or 9), and deliver the test to their fitness center [8]. Dedicated study personnel (nurses and medical doctors) provided onsite collection of all test kits, and facilitated self- sampling onsite for those who needed a personal explanation of the procedure on June 8 and 9, 2020. The fitness centers remained open for individuals in the training arm until June 15, but remained closed for the no-training arm. We also offered SARS-CoV-2 RNA testing to all fitness center employees who worked at the centers during the study period.

Transmission and contact tracing of individuals positive for SARS-CoV-2 RNA were performed by trained personnel from the Norwegian Institute of Public Health.

### Clinical endpoint assessment

On June 15, 2020 (3 weeks after study start), we retrieved all admissions and outpatient contacts for all somatic diagnoses (International Classification of Diseases, Tenth Revision); ICU (intensive care unit) admissions, ventilator treatment, and death for all participants from the trial area hospital databases. Norway has a public, single-payer hospital system with full coverage of data for all individuals. For individuals with diagnoses which may relate to Covid-19, we contacted physicians at the respective hospitals for details to investigate if the contact was related to Covid-19.

### SARS-CoV-2 antibody testing

Thirty days after study start (on June 24, 2020), all study participants who had provided a SARS-CoV-2 RNA test were mailed a self-sampling kit for antibody testing. The participants were asked to return the dried blood spot card by mail in a prepaid envelope by June 30, 2020. Vitas Analytical Services, Oslo, Norway, prepared each sample, and analyses were performed by the Department of Immunology, Oslo University Hospital. Measurement of IgG (immunoglobulin G) antibodies was performed with a multiplex flow cytometric assay known as microsphere affinity proteomics (MAP) [9]. Participants with insufficient quality or quantity of the dried blood spot sample or with test results close to a predefined cut-off for positivity (“borderline” results), were asked to provide a venous serum sample for analysis on Roche’s platform for SARS-CoV2 antibodies (Elecsys® Anti-SARS-CoV-2). Details are provided in the Supplementary Appendix.

Pre-intervention testing of antibodies was not performed because it was too resource and time-demanding, and because any risk of imbalance was deemed to be small due to the randomized design and the large number of included individuals in the trial.

### Outcome timing

The timing of the primary and secondary outcomes were based on estimated times from exposure to PCR positivity of 5 days [10], taking into account shorter incubation times than for symptomatic patients in a clinical testing because we tested all individuals, regardless of clinical symptoms. Antibody testing was performed 30 days after study start to maximize the likelihood of detection of infected individuals.

### Population data on Covid-19

From publicly available sources by the Norwegian Institute of Public Health (www.fhi.no) and the Norwegian Directorate of Health (www.helsedirektoratet.no), we retrieved data on number and rates of SARS-CoV-2 positive individuals, hospital admissions, intensive care treatment and death due to Covid-19 in Oslo during the study period.

During the study period, the Norwegian Institute of Public Health had the following recommendation for Covid-19 testing in the community: Immediate testing of persons with acute respiratory infection with fever, cough or dyspnoea belonging to one of the following groups (in prioritized order): Persons in need of hospital admission; residents in nursing homes or other health institutions; health care workers working in close contact with patients; persons aged above 65 or adults with chronic diseases; persons in quarantine due to close contact with a confirmed SARS-CoV-2-positive case or after travelling abroad. All other persons were advised to wait 48 h after symptom onset before considering testing, and persons without symptoms were not advised testing.

### Statistical analysis

We assumed non-inferiority of training versus no-training with regard to SARS-CoV-2 RNA positivity and hospital admission. Based on the most recent update of Covid-19 before the start of the trial from the Norwegian Institute of Public Health (May 11, 2020) and a rate of asymptomatic Covid-19 of at least 50% of those infected in the trial population, we assumed that 1% in each arm would test positive for SARS-CoV-2 RNA after 2 weeks. We defined the smallest meaningful absolute difference for SARS-CoV-2 transmission to be 1% between the two arms. Thus, the non-inferiority margin would be 1% for the training arm as compared to the no-training arm. For a power of 90% with an alpha of 0.05, we planned to include at least 1696 individuals in each arm.

The non-inferiority margin of 1% was an absolute difference within predefined boundaries of absolute risk of Covid-19 infection of up to 5%. Thus, any absolute difference of below 1% between the two groups with absolute rates of infection of 5% or lower was deemed non-inferior in the trial protocol. Further power calculations for transmission rates and hospital admission are provided in the Protocol.

The primary analytic approach of the trial follows the intention-to-treat (ITT) principle including all eligible individuals who were randomized and did not withdraw consent before start of intervention. The analyses of SARS-CoV-2 antibody testing was restricted to those who received the test kit. We compared the risk differences for the trial endpoints between the arms stratified by fitness center, using internal weights. Due to small numbers, we did not perform significance testing for all diagnosis sub-groups (Table 2). Analyses were performed using Stata Statistical Software release 16.

### Data completeness

We estimated the potential impact of non-complete follow-up for SARS-CoV-2 RNA testing and antibody testing assuming the following regarding positivity rate among those not compliant with testing 1) similar positivity rate of RNA tests as the average positivity rate of antibody tests for everyone who performed the antibody test; 2) similar positivity rate of RNA tests as the positivity rate of antibody tests in the respective randomization arm (training and no-training); 3) we estimated the number of participants not tested that would have to be positive in the training arm before the upper bound of the 95% confidence interval crosses the non-inferiority margin of 1%.

## Results

**Fig. 1:**
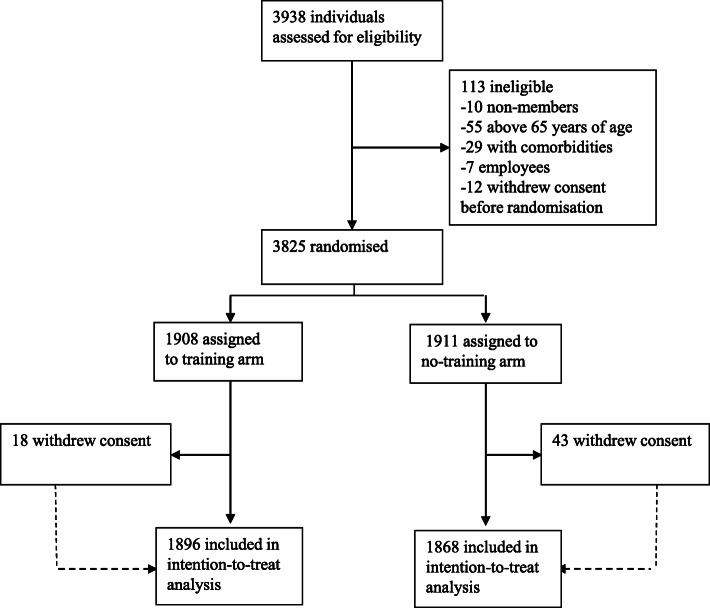
Study flowchart

Almost 67,000 fitness center members were approached between May 15 and May 24, 2020. Randomization of eligible individuals took place successively between May 20 and May 25, 2020, and the fitness centers were opened for participants randomized to the training arm on May 22, 2020. In total, 3938 individuals signed up for the trial online and provided written consent. Of these, 113 were ineligible and 3825 individuals were randomized. Sixty-one withdrew consent before start of the intervention, and thus 3764 individuals are included in the intention-to-treat analyses; 1896 in the training arm and 1868 in the no-training arm (Fig. 1). Participant characteristics shows that the arms were well-balanced (Table 1).

**Table 1:** Characteristics of the trial participants in the training and no-training arms, and of the employees working at the fitness centers during the trial. All data in numbers (%)

	Total	Training arm	No-Training arm
Trial participants	N	N (%)	N (%)
Individuals	3764	1896 (50.4)	1868 (49.6)
Sex			
Women	1929	974 (51.4)	955 (51.1)
Men	1835	922 (48.6)	913 (48.9)
Age at enrolment			
18–20 years	91	46 (2.4)	45 (2.4)
21–30 years	1278	643 (33.9)	635 (34.0)
31–40 years	1113	564 (29.7)	549 (29.4)
41–50 years	709	366 (19.3)	343 (18.4)
51–60 years	478	236 (12.4)	242 (12.0)
61–65 years	95	41 (2.2)	54 (2.9)
Training activity ^1^			
0 times		345 (18.2)	1868 (100)
1–2 times		314 (16.6)	0
3–5 times		435 (22.9)	0
6–10 times		464 (24.5)	0
More than 10 times		221 (11.7)	0
Employees			
Individuals	81		
Sex			
Women	56		
Men	25		
Age at enrolment			
18–20 years	3		
21–30 years	19		
31–40 years	24		
41–50 years	20		
51–60 years	14		
61–65 years	0		

^1^Times trained at fitness center during study period (data were available from four of the five facilities)

### Covid-19 in Oslo during the trial

The trial area was the city of Oslo with a population of around 690,000 [11]. During the first 2 weeks of the trial (from May 25 to June 7, 2020), 4410 individuals in Oslo were tested for SARS-CoV-2 RNA outside the trial [12]. The number of new cases was 105; 24 in the first week and 81 in the second week of the trial (rates per 100,000: 3.5 in first week, 11.7 in second week) [12]. The daily number of patients who were hospitalized in Oslo due to Covid-19 decreased gradually during the trial period, from 35 patients on May 22 to 21 patients on June 8, 2020.

### Training activity

Among individuals randomized to training, the majority (81.8%) trained at least once at the facility, and 38.5% trained six times or more (Table 1).

### SARS-CoV-2 RNA testing

**Fig. 2:**
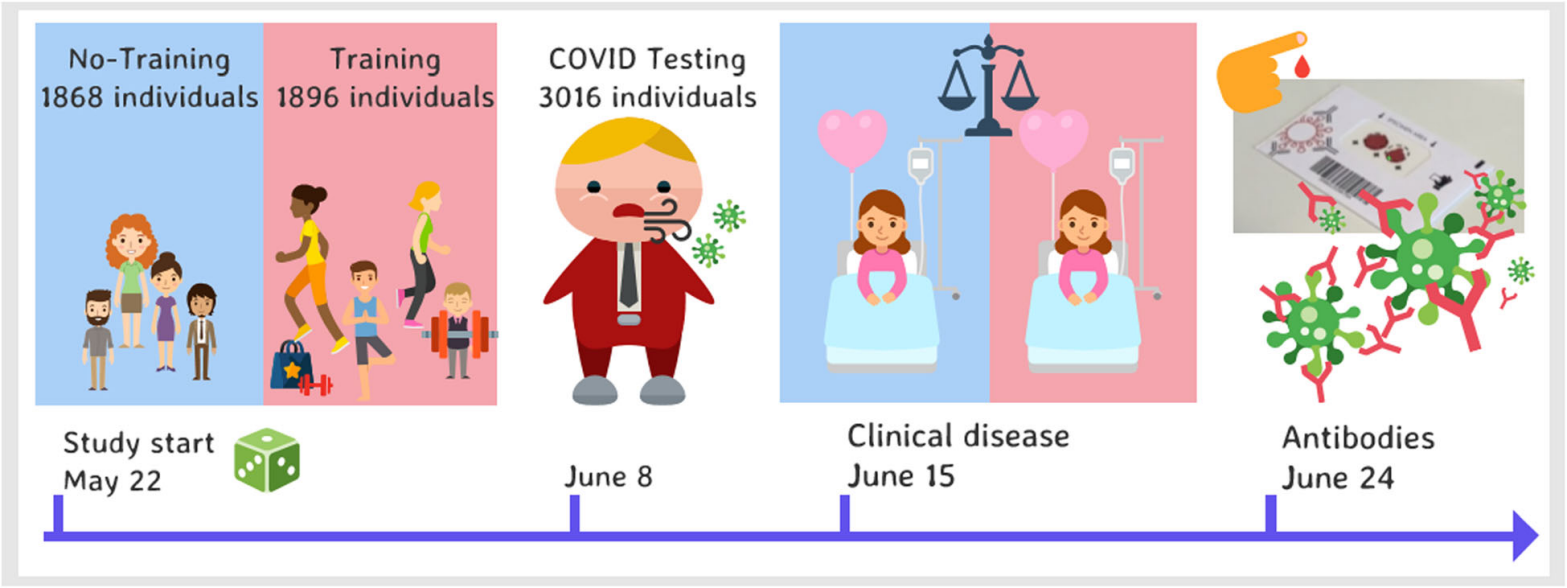
Study flowchart and graphical abstract (Figure developed by the authors using Venngage.com with license to use, reproduce and distribute worldwide)

**Fig. 3:**
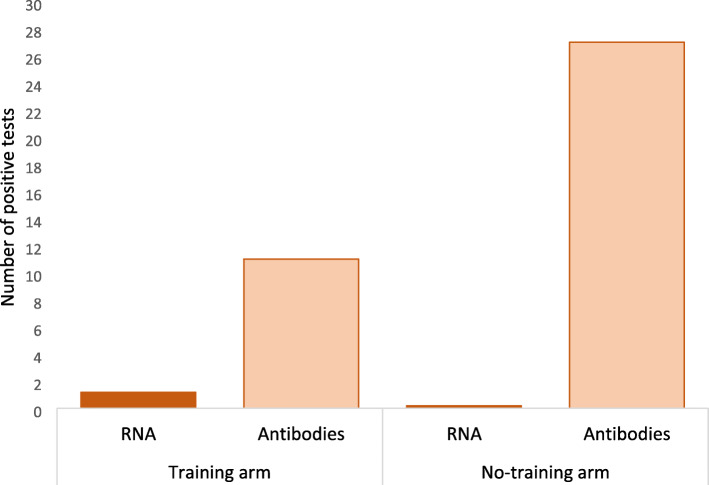
Number of positive tests for SARS-CoV-2 RNA and antibodies (IgG) in the training and no-training arm

After two weeks 3016 participants performed sampling for SARS-CoV-2 RNA (88.7% in the training arm; 71.4% in no-training arm). There was one positive test; in an individual randomized to the training arm and no positive tests in the no-training arm (risk difference 0.053%; 95%CI − 0.050 to 0.156%) (Table 2, Figs. 2 and 3). Transmission and contact tracing for the case revealed that the individual did not use the fitness center during the trial period until the day of the sampling, but had been present at the workplace where two other individuals had tested positive for SARS-CoV-2 RNA shortly before the participant tested positive in the trial. Thus, transmission was likely unrelated to the trial intervention, and there was no further transmission during trial intervention related to the case.

**Table 2:** SARS-CoV-2 RNA, clinical disease and SARS-CoV-2 RNA antibodies (IgG) in the training and no-training arms. All data in numbers (%)

	Total (3,764 individuals)	Training arm (1,896 individuals)	No-training arm (1,868 individuals)	*P*-value^4^
**SARS-CoV-2 RNA tests**	3,016 (80.1)	1,682 (88.7)	1,334 (71.4)	
**Positive SARS-CoV-2 RNA**	1	1^1^ (0.05)	0	0.32
**Covid-19 related hospital admissions**	0	0	0	
**Non-Covid-19 related hospital admissions**	6 (0.16)	4 (0.21)	2 (0.11)	0.42
Cardiovascular	1	1	0	
Gastroenterology	1	0	1	
Surgery^2^	3	3	0	
Gynecology	1	0	1	
**Covid-19 outpatient contacts**	0	0	0	
**Non-Covid-19 outpatient contacts**	106 (2.8)	48 (2.5)	58 (3.1)	0.29
Surgery^2^	46	20	26	
Gynecology	15	8	7	
Endocrinology/nephrology	12	6	6	
Cardiovascular	4	3	1	
Pulmonology	3	2	1	
Gastroenterology	6	0	6	
Dermatology	3	2	1	
Oncology	9	3	6	
Neurology	8	4	4	
**SARS-CoV-2 Antibody tests performed**	2516	1404	1112	
**Positive SARS-CoV-2 antibodies**	38 (1.5^3^)	11 (0.8^3^)	27 (2.4^3^)	0.008

^1^Infection not related to training activity

^2^Surgery includes orthopedics and Ear-Nose-Throat

^3^Percentage of SARS-CoV-2 antibody tests performed

^4^All statistical test are performed on the intention-to-treat level

### Covid-19 and associated disease

A total of 106 outpatient contacts for somatic disease in the hospitals serving the trial area were registered for 106 (2.8%) participants (Table 2). There were six hospital admissions among participants; four in the training arm and two in the no-training arm. Five of the hospital admissions were unrelated to any Covid-19 associated condition. One patient was admitted with pulmonary embolism. We contacted the attending physician who after blinded chart review ruled out that the condition was related to Covid-19. Thus, no trial participants had hospital admissions or outpatient visits related to Covid-19 (Table 2).

### SARS-CoV-2 antibody testing

Kits for SARS-CoV-2 antibody testing were mailed to all participants who had provided a SARS-CoV-2 RNA test; 1682 in the training arm and 1334 in the no-training arm. The return rate was 83.4% in both the training arm (1404 individuals) and the no-training arm (1112 individuals). In total, 11 individuals in the training arm (0.8% of those tested) and 27 in the no-training arm (2.4% of those tested) tested positive for SARS-CoV-2 antibodies (risk difference − 0.87%; 95% CI − 1.52% to − 0.23%, *p* = 0.001) (Figs. 2 and 3). Details about the antibody testing are provided in the Supplementary Appendix, Fig. S1.

### Potential impact of non-complete data

More women were compliant with SARS-CoV2 testing in the training arm as compared to the no-training arm, and compliant individuals were somewhat younger in the training arm compared to the non-training arm (Supplementary Appendix, Table S1). The age, and sex distribution of those with positive antibody tests was similar and there was no difference in hospital contacts between the study arms (Supplementary Appendix, Table S2).

Sensitivity analyses confirmed the robustness of the primary analyses: Assuming that the positivity rate among those not tested is similar to the average positivity rate of antibody tests for everyone who took the test, 4 participants in the training arm (11,3% not tested) and 8 participants in the no-training arm (28,6% not tested) would have tested positive (risk difference − 0.22, 95% confidence interval − 0.58 to 0.14%). Assuming a similar positivity rate of RNA tests among those not tested as the positivity rate of antibody tests in the respective randomization arm (training and no-training), 2 participants in the training arm and 13 participants in the no-training arm, would have tested positive (risk difference − 0.59, 95% confidence interval − 0.996% to − 0.19%).

14 participants in the training arm and none in the no-training arm had to test positive before the upper bound of the 95% confidence interval crossed the predefined non-inferiority margin of 1% (risk difference 0.69, 95% confidence interval 0.32 to 1.06%).

### Fitness center employee assessment

Out of 81 employees who worked at the fitness centers during the trial period, 76 (93.3%) were tested for SARS-CoV-2 RNA. None were positive. 58 employees provided antibody tests and none were positive.

## Discussion

Our trial showed no incremental risk of being infected with SARS-CoV-2 in fitness centers with strict enforcement of comprehensive hygiene and physical distancing measures, as compared to community exposure. There was no increase in SARS-CoV-2 antibodies related to re-opening of fitness centers. The difference in SARS-CoV-2 RNA test positivity between the training and no-training arms was 0.05% (one versus zero cases), well below the predefined non-inferiority margin of an absolute difference of 1%.

During the Covid-19 pandemic, many countries introduced closure of important societal activities to contain virus transmission, and by emergency law, all fitness centers were closed in Norway. However, if virus containment, including contact tracing and quarantine, hand hygiene and personal physical distancing measures are sufficient to prevent virus spread, closures could be avoided. Our trial sought to test if closure of fitness centers was needed in Oslo, Norway, in May and June 2020. If hygienic and distancing measures could be achieved, we assumed it would be safe to re-open fitness centers. The trial did not test what would happen if fitness centers open during the pandemic with no hygienic and distancing measures.

For the purpose of the trial, the research group and the Norwegian fitness center association (Virke Training) established national mitigation guidelines for hygiene and physical distancing for fitness centers in Norway in collaboration with the Norwegian Institute of Public Health. The guidelines were used in the trial and enforced by employees at the facilities at all times.

The primary concern with Covid-19 is serious disease, measured as hospital admission, need for ventilator support, and death. As a surrogate, positivity for SARS-CoV-2 RNA is often used. However, high SARS-CoV-2 RNA test positivity in individuals or groups of individuals is not necessarily a surrogate for severity of Covid-19 in a population, because SARS-CoV-2 infected individuals who do not become seriously ill or who do not transmit the disease to others who become seriously ill, may contribute to achieve immunity in the population and thus contain the disease. Therefore, we measured both SARS-CoV-2 RNA positivity and incidence of serious Covid-19 or associated disease to understand the relationship of the surrogate outcome with the clinically significant disease outcomes. As our results show, there was no increase in Covid-19-related disease due to the opening of fitness centers.

Our trial was limited by the low number of events in both arms. Only one individual tested positive for SARS-CoV-2 RNA, and there was no serious Covid-19 among participants during the trial. As shown, there was indeed transmission of SARS-CoV-2 in Oslo during the study period, with both new cases and patients outside of the trial admitted to hospital with Covid-19. Our results may reflect the low risk of virus transmission and serious Covid-19 in healthy individuals without Covid-19 symptoms or risk factors, who were those who participated in the trial. Although trial participants may be highly motivated to exercise at their fitness center, so are many members at such centers, and we have no indication that our trial population is not representative of many users of fitness centers. Thus, the results may be applied to other regions and countries with similar community prevalence of Covid-19 [13].

The rate of new positive tests outside the trial in Oslo during the trial period was not substantially different to that of many states and counties in the United States reported in the same period (e.g. positive test rate per 100,000 individuals in the week of June 15 to 21, 2020 was 13 in Maine, 25 in New Jersey, and 22.5 in Massachusetts) [13]. It is, however, unclear if our findings would apply to areas with higher SARS-CoV-2 and Covid-19 incidence rates.

Although we timed the outcome testing for RNA PCR and antibodies according to best estimates for SARS-CoV-2 incubation periods, we cannot exclude that some individuals who may have been infected late during the intervention were not detected by RNA PCR testing. Also, it is possible that some individuals who had been exposed to Covid-19 had late antibody responses that were not picked up by our testing after 30 days. However, any large differences between treatment arms would have been apparent with antibody testing or hospital admission, if present.

Finally, it may be argued that the observed difference in antibodies between the two treatment arms may be due to differences at the start of the trial. We cannot rule this out, but find it unlikely because the concept of randomization includes reducing imbalances at baseline, especially in trials with several thousand individuals.

Our sample size was based on estimates from prevalence testing in the community for SARS-CoV-2. Most individuals in community testing had clinical signs or symptoms indicative of Covid-19. Thus, in accordance with evidence from population sampling in Iceland [14], we assumed considerably higher SARS-CoV-2 RNA rates in our sampling of individuals with no symptoms.

Compliance with SARS-CoV-2 RNA testing was higher in the training arm (89%) than in the no-training arm (71%). However, disease endpoints in the trial were gathered through complete hospital registries and are not prone to self-reporting bias. Also, the number of individuals who withdrew consent after randomization was small (18 in the training arm and 43 in the no-training arm). Finally, sensitivity analyses investigating effect of missing data confirm the robustness of the estimates.

We did not observe more individuals with SARS-CoV-2 antibodies amongst individuals in the training arm, supporting the results of the RNA testing and the data on Covid-19. In fact, there were significantly more individuals with detected SARS-CoV-2 antibodies in the no-training arm than in the training arm. The trial was not designed to establish any protective effect of training against Covid-19 and these results should be interpreted with caution. A possible explanation for the difference may be alternative exercise patterns in uncontrolled environments in the individuals in the no-training arm who did not have access to a fitness center. This deserves further study.

## Conclusions

Provided good hygiene and physical distancing measures, there was no increased infection risk of SARS-CoV-2 at fitness centers in Oslo, Norway in May/June 2020. The Norwegian government indeed allowed re-opening of all facilities as of June 15, 2020, provided that the hygienic and social distancing measures applied in the trial could be followed. It is important to perform randomized implementation and de-implementation of societal measures with large potential harms and burden for individuals and the population. Our study show that it is feasible to apply rigorous randomized testing of public health measures during an ongoing disease outbreak, according to the principles of rapid-cycle randomized implementation for health care services [15, 16].

## Supplementary Information

**Additional file 1.** A Randomized Trial of Covid-19 Transmission in Fitness centers

## Data Availability

The individual patient datasets generated during the current study are not publicly available, but anonymized datasets may be available from the corresponding author on reasonable request.

## Abbreviations

CONSORT: Consolidated Standards of Reporting Trials
Covid-19: Coronavirus disease of 2019
ICU: Intensive care unit
IgG: Immunoglobulin G
RNA: Ribonucelic acid
RT-PCR: Reverse transcription-polymerase chain reaction
SARS-CoV-2: Severe acute respiratory syndrome coronavirus 2

## References

[1] WHO. WHO Director-General's opening remarks at the media briefing on COVID-19 - 11 March 2020. Available from: https://www.who.int/director-general/speeches/detail/who-director-general-s-opening-remarks-at-the-media-briefing-on-covid-19---11-march-2020. Accessed 13 Mar 2020.

[2] Flaxman S, Mishra S, Gandy A, Unwin HJT, Mellan TA, Coupland H, Whittaker C, Zhu H, Berah T, Eaton JW, Monod M, Perez-Guzman PN, Schmit N, Cilloni L, Ainslie KEC, Baguelin M, Boonyasiri A, Boyd O, Cattarino L, Cooper LV, Cucunubá Z, Cuomo-Dannenburg G, Dighe A, Djaafara B, Dorigatti I, van Elsland SL, FitzJohn RG, Gaythorpe KAM, Geidelberg L, Grassly NC, Green WD, Hallett T, Hamlet A, Hinsley W, Jeffrey B, Knock E, Laydon DJ, Nedjati-Gilani G, Nouvellet P, Parag KV, Siveroni I, Thompson HA, Verity R, Volz E, Walters CE, Wang H, Wang Y, Watson OJ, Winskill P, Xi X, Walker PGT, Ghani AC, Donnelly CA, Riley S, Vollmer MAC, Ferguson NM, Okell LC, Bhatt S, Imperial College COVID-19 Response Team (2020). Estimating the effects of non-pharmaceutical interventions on COVID-19 in Europe. Nature.

[3] Ioannidis JPA. A fiasco in the making? As the coronavirus pandemic takes hold, we are making decisions without reliable data. STAT, March 17, 2020. Available from: https://www.statnews.com/2020/03/17/a-fiasco-in-the-making-as-the-coronavirus-pandemic-takes-hold-we-are-making-decisions-without-reliable-data/?fbclid=IwAR3Ss4Qb8MM3EN_BxPFCetD2uF4usls_IP5AV1Iy9E0d54IpfryZMyIgB9g. Accessed 21 June 2020.

[4] Norwegian Ministry of Health and Care Services (2020). Forskrift om smitteverntiltak mv. ved koronautbruddet (covid-19 forskriften).

[5] Norwegian Ministry of Health and Care Services (2020). Forskrift om endring i forskrift om smitteverntiltak mv. ved koronautbruddet (covid-19 forskriften).

[6] Helsingen LM, Refsum E, Gjøstein DK, et al. The COVID-19 pandemic in Norway and Sweden – threats, trust, and impact on daily life: a comparative survey. BMC Public Health. 2020;20:1597. 10.1186/s12889-020-09615-3. 10.1186/s12889-020-09615-3 PMC7582026 33097011

[7] Norwegian Institute of Public Health. Risk groups and their relatives - advice and information 2020. Available from: https://www.fhi.no/en/op/novel-coronavirus-facts-advice/facts-and-general-advice/risk-groups%2D%2D-advice-and-information/. Accessed 15 May 2020.

[8] Juvet LK, Lauvrak V (2020). Saliva sample for testing SARS-CoV-2 infection – a rapid review.

[9] Holter JC, Pischke SE, de Boer E (2020). Systemic complement activation is associated with respiratory failure in COVID-19 hospitalized patients. Proc Natl Acad Sci.

[10] Sethuraman N, Jeremiah SS, Ryo A (2020). Interpreting diagnostic tests for SARS-CoV-2. JAMA.

[11] Statistics Norway (2020). Population statistics Oslo.

[12] Norwegian Institute of Public Health (2020). Covid-19 weekly report.

[13] Johns Hopkins University of Medicine Coronavirus Resource Center. Available from: https://coronavirus.jhu.edu/testing/individual-states/usa. Accessed 24 June 2020.

[14] Gudbjartsson DF, Helgason A, Jonsson H, Magnusson OT, Melsted P, Norddahl GL, Saemundsdottir J, Sigurdsson A, Sulem P, Agustsdottir AB, Eiriksdottir B, Fridriksdottir R, Gardarsdottir EE, Georgsson G, Gretarsdottir OS, Gudmundsson KR, Gunnarsdottir TR, Gylfason A, Holm H, Jensson BO, Jonasdottir A, Jonsson F, Josefsdottir KS, Kristjansson T, Magnusdottir DN, le Roux L, Sigmundsdottir G, Sveinbjornsson G, Sveinsdottir KE, Sveinsdottir M, Thorarensen EA, Thorbjornsson B, Löve A, Masson G, Jonsdottir I, Möller AD, Gudnason T, Kristinsson KG, Thorsteinsdottir U, Stefansson K (2020). Spread of SARS-CoV-2 in the Icelandic population. N Engl J Med.

[15] Horwitz LI, Kuznetsova M, Jones SA (2019). Creating a learning health system through rapid-cycle, randomized testing. N Engl J Med.

[16] Kalager M, Bretthauer M (2020). Improving cancer screening programs. Science.

