# Supplementary material for: Covid-19 transmission in fitness centers in Norway - a randomized trial
Source: BMC Public Health. 2021 Nov 16;21:2103. doi: 10.1186/s12889-021-12073-0 (PMC8595959; doi:10.1186/s12889-021-12073-0)
Supplement: Supplementary file 1 — Additional file 1. A Randomized Trial of Covid-19 Transmission in Fitness centers [file 12889_2021_12073_MOESM1_ESM.docx]

|  |
| --- |
| Supplementary Appendix |
| **A Randomized Trial of Covid-19 Transmission in Fitness centers** |

**Table of Contents**

[**List of investigators and committees** 2](#_Toc63681374)

[TRAiN Study Group 2](#_Toc63681375)

[Data Safety and Monitoring Committee 2](#_Toc63681376)

[Participating fitness centers 2](#_Toc63681377)

[**Infection preventive measures at participating fitness centers** 3](#_Toc63681378)

[**Details of SARS-CoV-2 antibody testing** 5](#_Toc63681379)

[Preparation of samples 5](#_Toc63681380)

[Analyses 5](#_Toc63681381)

[**Figures** 7](#_Toc63681382)

[Figure S1: Flow chart of antibody (IgG) testing 7](#_Toc63681383)

[**Tables** 8](#_Toc63681384)

[Table S1: Number and amount (%) of participants tested for SARS-CoV-2 RNA in the training and the no-training arm by sex, age, and hospital contacts. 8](#_Toc63681385)

[Table S2: Number and amount (%) of participants tested for SARS-CoV-2 antibodies in training and no-training arm by sex, age, admission and hospital contact 9](#_Toc63681386)

[**References** 10](#_Toc63681387)

# **List of investigators and committees**

## TRAiN Study Group

Lise M. Helsingen, Magnus Løberg, Erle Refsum, Dagrun Kyte Gjøstein, Paulina Wieszczy, Frederik E. Juul, Ishita Barua, Henriette C. Jodal, Magnhild Herfindal, Yuichi Mori, Michael Bretthauer, Mette Kalager, Anita Aalby, Madeleine Berli, Siv Furholm and Anne-Lise Horvli (Clinical Effectiveness Research Group, University of Oslo and Oslo University Hospital, Norway); Line Norum (STOLT Training, Oslo, Norway); Halvor Lauvstad and Judit Somogyi (EVO Training, Oslo, Norway); Alexander Myers, Tonje Poulsson and Wenche Evertsen (SATS Training, Oslo, Norway); Hilde Sandvoll and Kjersti Oppen (Virke Training, Oslo, Norway); Ørjan Olsvik (Institute of Medical Biology, Norwegian Arctic University, Tromsø, Norway); Solveig Jore and Atle Fretheim (Norwegian Institute of Public Health, Oslo, Norway); Fridtjof Lund-Johansen (Department of Immunology, Oslo University Hospital, Oslo, Norway)

## Data Safety and Monitoring Committee

Katja Fall MD, PhD, Örebro University, Sweden

Dag Berild MD, PHD, Oslo University Hospital

## Participating fitness centers

SATS Sjølyst (SATS Norway Inc., Oslo, Norway)

SATS CC Vest (SATS Norway Inc., Oslo, Norway)

STOLT Stovner (STOLT Trening Inc, Oslo, Norway)

STOLT Rommen (STOLT Trening Inc, Oslo, Norway)

EVO Bryn (EVO Fitness Group Inc, Oslo, Norway).

# **Infection preventive measures at participating fitness centers**

| **Standard for COVID-19 infection prevention measures in fitness centers during the TRAiN-study**  Collaboration between Virke Trening, the Norwegian Institute of Public health (NIPH) and the Clinical Effectiveness Research Group (UiO and OUS) | |
| --- | --- |
| General | - If you have symptoms compatible with COVID-19 you should *not* be present at the fitness center. - Focus on good hygiene: Keep the fitness center clean, perform frequent hand wash and cough in your elbow if needed. Generally avoid touching your face. - Wash hands and clean training equipment before and after use with utensils provided by the fitness center. Fitness center employees performs thorough cleaning of the facility and training equipment at closure each day and in between during opening hours, depending on frequency of use and type of equipment. - Keep distance – No physical contact with others at the facility. |
| Distance | - Keep at least 1 meter distance between you and others at all times. - When performing hard intensity activities keep a distance of at least 2 meters between you and others. |
| Group classes | - Number of people that could attend depends on the size of the gym as well as the associated changing rooms, showers and toilets. - High intensity activities: at least 2 meters of distance between persons. - At least 1 meter of distance between persons at all times. - Avoid queuing: Make sure group classes do not start and stop at the same time. - Keep a minimum of at least 15 minutes between each group class. - A washbasin with soap or hand disinfection should be available in the gym. - Classes including physical contact between participants or participants and instructors are not allowed. - Make sure all instructors have personal microphones, or that shared microphones are disinfected appropriately. |
| Personal training | - Remind about good hygiene and physical distance - Wash hands before and after training, clean training equipment after use. |
| Changing rooms | - Changing rooms, showers and sauna could be kept open if it is possible to keep at least 1 meter distance between users at all times. - During the TRAiN study changing rooms will be open, while showers and saunas will be closed. |
| Cleaning | - The fitness center should be cleaned before re-opening. - Create lists of what should be cleaned and with what frequency. - Provide extra cleaning of particularly frequently touched surfaces (eg door handles, card readers, washbasin batteries) and otherwise if necessary - Have disinfectant readily available in strategic places in the facilities (reception, at booking station, changing rooms and toilets) - Frequent refilling at all hygiene stations - Have disinfectant available at water taps/springs used for drinking or refilling bottles - Remove lid on trash cans - Wash appliances with cloth and soapy water regularly |
| Operation of the facilities | - Make an overview of the maximum number of members who can train at the same time to maintain 1 and 2 meters distance between the members as well as capacity for toilets, showers and changing rooms. |
| Communication | - Reminders of infection preventive measures - information online and via posters in the facilities - Inform about changes related to the training offer (distance, etc.) |
| Childcare | - If it is difficult to maintain at least 1 meter between the children, the childcare facilities must be kept closed. |

# **Details of SARS-CoV-2 antibody testing**

## Preparation of samples

Participants were mailed a self-sampling kit containing a Whatman 903 paper card for dried-blood spot sampling (Vitas Analytical Services, Oslo, Norway), and returned it by mail. Vitas Analytical Services, Oslo, Norway prepared each sample for analysis by robotized 3.2mm punching into 96-well storage plates with 150μl of phosphate-buffered saline and 1% sodium azide. The plates were closed and sealed before transported to the Department of Immunology, Oslo University Hospital, for analysis.

## Analyses

Ten microliters of eluate was added to 90 μl of assay buffer (PBS with 1% Tween 20, 10μg/ml d-biotin and 10mg/ml neutravidin). Measurement of IgG antibodies to Nucleocapsid and the receptor-binding domain (RBD) of Spike1 from SARS-CoV-2 was next performed with a multiplex flow cytometric assay known as microsphere affinity proteomics (MAP), as previously described (1). Samples were considered positive if they contained antibodies to both nucleocapsid and RBD. A reference panel containing samples from 63 individuals with PCR-confirmed SARS-CoV-2 infection and 86 pre-pandemic samples were used to set the cut-off. With a cut-off set to obtain a specificity of 100%, the sensitivity was 84%. Participants with insufficient quality or quantity of the dried blood spot sample for testing, and participants with test results close to the predefined cut-off for positivity (“borderline” results), were asked to provide a venous serum sample for analysis on Roche’s platform for SARS-CoV2 antibodies (Elecsys® Anti-SARS-CoV-2).

# **Figures**

## Figure S1: Flow chart of antibody (IgG) testing


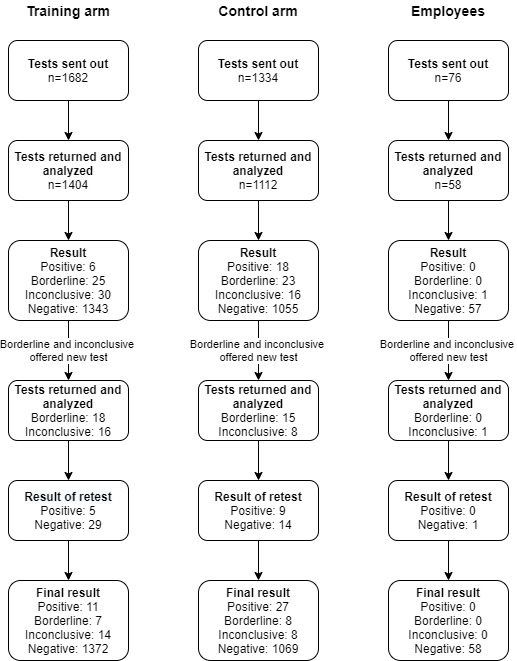


# **Tables**

## Table S1: Number and proportion (%) of participants tested for SARS-CoV-2 RNA in the training and the no-training arm by sex, age, and hospital contacts.

|  |  | Training arm | | | | No-training arm | | | |
| --- | --- | --- | --- | --- | --- | --- | --- | --- | --- |
|  |  | Tested | | Not tested | | Tested | | Not tested | |
|  |  | n | % | n | % | n | % | n | % |
| Participants | | 1683 |  | 213 |  | 1334 |  | 534 |  |
| Sex |  |  |  |  |  |  |  |  |  |
|  | Female | 856 | 88% | 118 | 12% | 744 | 78% | 211 | 22% |
|  | Male | 827 | 90% | 95 | 10% | 590 | 65% | 323 | 35% |
| Age group |  |  |  |  |  |  |  |  |  |
|  | 18-19 | 41 | 89% | 5 | 11% | 23 | 51% | 22 | 49% |
|  | 20-29 | 536 | 83% | 107 | 17% | 386 | 61% | 249 | 39% |
|  | 30-39 | 499 | 88% | 65 | 12% | 392 | 71% | 157 | 29% |
|  | 40-49 | 339 | 93% | 27 | 7% | 279 | 81% | 64 | 19% |
|  | 50-59 | 228 | 97% | 8 | 3% | 208 | 86% | 34 | 14% |
|  | 60-65 | 40 | 98% | 1 | 2% | 46 | 85% | 8 | 15% |
| Hospital contact | |  |  |  |  |  |  |  |  |
|  | No | 1625 | 89% | 205 | 11% | 1289 | 72% | 512 | 28% |
|  | Yes | 58 | 88% | 8 | 12% | 45 | 67% | 22 | 33% |

## Table S2: Number and proportion (%) of participants tested for SARS-CoV-2 antibodies in training and no-training arm by sex, age, admission and hospital contact

|  |  | Training group | | | | Control group | | | |
| --- | --- | --- | --- | --- | --- | --- | --- | --- | --- |
|  |  | Negative/not tested | | Positive | | Negative/not tested | | Positive | |
|  |  | n | % | n | % | n | % | n | % |
| Participants | | 1885 |  | 11 |  | 1841 |  | 27 |  |
| Sex |  |  |  |  |  |  |  |  |  |
|  | Female | 968 | 99% | 6 | 1% | 940 | 98% | 15 | 2% |
|  | Male | 917 | 99% | 5 | 1% | 901 | 99% | 12 | 1% |
| Age group |  |  |  |  |  |  |  |  |  |
|  | 18-19 | 45 | 98% | 1 | 2% | 45 | 100% | 0 | 0% |
|  | 20-29 | 640 | 100% | 3 | 0% | 631 | 99% | 4 | 1% |
|  | 30-39 | 560 | 99% | 4 | 1% | 540 | 98% | 9 | 2% |
|  | 40-49 | 364 | 99% | 2 | 1% | 337 | 98% | 6 | 2% |
|  | 50-59 | 236 | 100% | 0 | 0% | 236 | 98% | 6 | 2% |
|  | 60-65 | 40 | 98% | 1 | 2% | 52 | 96% | 2 | 4% |
| Hospital contact | |  |  |  |  |  |  |  |  |
|  | No | 1820 | 99% | 10 | 1% | 1774 | 99% | 27 | 1% |
|  | Yes | 65 | 98% | 1 | 2% | 67 | 100% | 0 | 0% |

# **References**

1. Holter,JC, Pischke SE,  de Boer E et al. Systemic complement activation is associated with respiratory failure in COVID-19 hospitalized patients. PNAS 2020.  <https://doi.org/10.1073/pnas.2010540117>
